# Interfacial Characteristics and Metallurgical Mechanism of Sapphire/TC4 Alloy Joints Produced by Femtosecond Laser Welding

**DOI:** 10.3390/ma19204286

**Published:** 2026-10-11

**Authors:** Yue Zhao, Xiaoyang Wang, Jiangtao Liang, Sen Huang, Yinghao Cui, Quanqi Zuo, Shirun Guo, Lujun Cui

**Affiliations:** 1 School of Intelligent Mechatronic Engineering, Zhongyuan University of Technology, Zhengzhou 451191, Chinayh_cui@zut.edu.cn (Y.C.); laser@zut.edu.cn (S.G.);; 2 Shougang Research Institute of Technology, Shougang Group Co., Ltd., Beijing 100043, China; 3 School of Aero Engine, Zhengzhou University of Aeronautics, Zhengzhou 450046, China; 4 State Key Laboratory of High Performance & Advanced Welding Materials, China Academy of Machinery Zhengzhou Research Institute of Mechanical Engineering Co., Ltd., Zhengzhou 450001, China

**Keywords:** sapphire, TC4 alloy, femtosecond laser welding, interfacial metallurgical mechanism

## Abstract

Femtosecond laser welding of sapphire to TC4 (Ti–6Al–4V) alloy was conducted under atmospheric conditions, and the interfacial characteristics, metallurgical reaction mechanism, and the effect of laser power on joint strengthening were systematically investigated. The results revealed that the femtosecond laser induced transient high temperatures, elemental intermixing, and interfacial metallurgical reactions at the sapphire/TC4 alloy interface, producing an interfacial compositional transition zone with a maximum measured width of 5.72 μm after rapid cooling. Reaction products including TiO, TiO_2_, and Al_3_Ti were identified by XRD, while EDS revealed interfacial redistribution of Ti, Al, and O. At a laser power of 8.4 W, the joint exhibited a relatively flat interfacial morphology and a lower apparent shear strength than that at 12.4 W. The highest mean apparent shear strength of 51.55 MPa was obtained at 12.4 W, which was associated with the characteristic wavy interfacial morphology that promoted crack deflection and energy dissipation. At 16.6 W, interconnected microcracks formed near the interface and within the sapphire substrate due to excessive heat input and thermal mismatch, and the apparent shear strength decreased to a level statistically comparable to that at 8.4 W.

## 1. Introduction

Sapphire (α-Al_2_O_3_) exhibited superior optical transmittance, mechanical performance, high-temperature stability, and wear resistance. Sapphire has been used in fields such as infrared windows, substrates for optoelectronic devices, aerospace protective components, and medical devices [1,2]. TC4 alloy is an engineering titanium alloy with high specific strength, excellent corrosion resistance and good biocompatibility, and is widely applied in aerospace, energy equipment and medical devices [3]. Benefiting from the outstanding comprehensive performance of sapphire/titanium alloy assemblies, the fabrication of high-quality dissimilar joints between the two materials has long garnered substantial industrial interest [4,5]. Accordingly, developing a joining method capable of simplifying processing conditions while guaranteeing robust interfacial bonding is of great practical significance [6].

The substantial differences in thermophysical properties between sapphire and TC4 alloy continued to pose considerable challenges to their welding [7]. Conventional ceramic–metal joining methods mainly include brazing, diffusion bonding and laser welding [8,9,10]. However, brazing and diffusion bonding had evident limitations, including long processing cycles, prolonged bulk heating and degradation of the base-material properties [11]. Laser welding has been applied to ceramic–metal joining because of its concentrated energy input, controllable processing path, and capability for localized heating. Continuous-wave laser irradiation involves a relatively long interaction time and high heat input, which may cause cracking of sapphire during welding [12]. In comparison, short-pulse lasers can reduce the total heat input and thereby lower the risk of cracking [13,14]. Moreover, as the laser pulse duration decreases, less energy is transferred to the lattice of sapphire through linear absorption and thermal conduction. The reduction in heat input facilitated the formation of a smaller molten pool and heat-affected zone, which reduced the risk of sapphire cracking [15,16].

Ultrafast lasers, possessing femtosecond-scale ultrashort pulse durations and ultrahigh peak powers, were capable of triggering intense nonlinear multiphoton absorption and plasma breakdown at the heterogeneous interfaces between transparent materials and metals [17,18,19]. The excellent spatial confinement allows laser energy to be precisely deposited within a micrometer-scale focal region, enabling localized melting and metallurgical bonding. This characteristic effectively avoids the formation of extensive heat-affected zones induced by linear absorption during conventional laser welding [20]. Existing investigations show that ultrafast laser microwelding is applicable to a variety of complex material systems, represented by glass–aluminum and glass–glass-ceramic combinations [21,22]. Direct microwelding of sapphire with metallic materials such as Fe-36Ni [23], Al [24], Cu [25], and stainless steel [26] has also been achieved. These studies have primarily focused on the influences of scanning speed, surface state and energy input on interfacial morphology, elemental transition zones and joint mechanical strength. The reported results indicate that jagged interfacial mechanical interlocking, localized elemental migration, and regulation of thermal deposition are important factors governing joint performance [27]. However, when Ti-based reactive materials are involved, the interfacial metallurgical behavior can become more complex. Pronounced elemental diffusion and interfacial reactions have been reported at Ti/Al_2_O_3_ interfaces [28]. Elemental interdiffusion across sapphire–metal interfaces has also been observed during femtosecond laser welding [29]. In Ti-containing ceramic–metal joints, ultrashort-pulse laser irradiation can promote elemental redistribution and the formation of interfacial reaction products [30,31]. When a transient liquid phase is present, interfacial reactions can affect not only phase formation but also the wetting and spreading behavior by altering the local chemistry, structure, and energetics of the solid–liquid interface. Such reaction-induced interfacial evolution and the associated liquid spreading are characteristic features of reactive wetting in high-temperature capillarity [32,33,34]. Accordingly, elemental redistribution, interfacial reaction, and transient liquid spreading are closely coupled during ceramic–metal joining. However, excessive heat input may intensify interfacial reactions and increase the susceptibility to interfacial defects and cracking. Furthermore, the substantial mismatch in thermal expansion coefficients between sapphire and titanium alloy can generate considerable residual thermal stress during cooling, which may be further aggravated under high heat input. The combined effects of interfacial reactions and thermal stresses can therefore promote microcrack formation and reduce the macroscopic load-bearing capacity of the joint.

In this paper, sapphire and TC4 alloy were directly joined using a femtosecond laser. Particular attention was paid to the joint morphology, interfacial characteristics, fracture behavior and strengthening mechanisms under different laser-power conditions. This study aimed to provide experimental evidence and mechanistic insights for the heterogeneous joining of transparent ceramics and reactive metals.

## 2. Materials and Methods

### 2.1. Materials

Single-crystal sapphire specimens with dimensions of 10 mm × 10 mm × 1 mm and a C-plane (0001) orientation were used in this study. TC4 (Ti–6Al–4V) alloy plates were cut into specimens with dimensions of 10 mm × 10 mm × 3 mm by wire electrical discharge machining. The commercial TC4 alloy plates were provided by Dongguan Jiabo Metal Materials Co., Ltd., Dongguan, China and were accompanied by quality certificate JS2023-311, confirming compliance with the GB/T 3621-2007 standard [35]. The certified chemical composition of the TC4 alloy is listed in Table 1, including an oxygen content of 0.16 wt%. The sapphire surfaces were double-sided polished to a surface roughness of Ra < 5 nm, and the TC4 alloy surfaces were ground using 1200-grit abrasive paper to achieve Ra < 400 nm. Before welding, both materials were ultrasonically cleaned in ethanol to remove surface contaminants and were subsequently assembled using a specially designed fixture. The fixture was used to maintain stable contact between the sapphire and TC4 alloy during welding. The applied clamping force was not independently measured or calibrated; therefore, it was not treated as a controlled quantitative process parameter in this study.

### 2.2. Femtosecond Laser Welding System

The welding experiments were conducted using a commercial ultrafast fiber laser system (HR-Femto-GN-20-15, Wuhan Huaray Precision Laser Co., Ltd., Wuhan, China). The customized system was capable of delivering infrared laser light at 1034 nm and green laser light at 532 nm. In this study, the infrared laser with a central wavelength of 1034 nm and a pulse duration of 211 fs was employed. The laser beam was delivered through a two-dimensional galvanometer scanning system (ZB2D14A1064-W, Shenzhen ZBTK Technology Co., Ltd., Shenzhen, China) and focused onto the sapphire/TC4 alloy interface using an F-theta lens with a focal length of 100 mm (SL-1064-70-100G, Nanjing Wavelength Opto-Electronic Science & Technology Co., Ltd., Nanjing, China). Welding experiments were conducted in ambient air at room temperature with no shielding gas applied.

**Figure 1:**
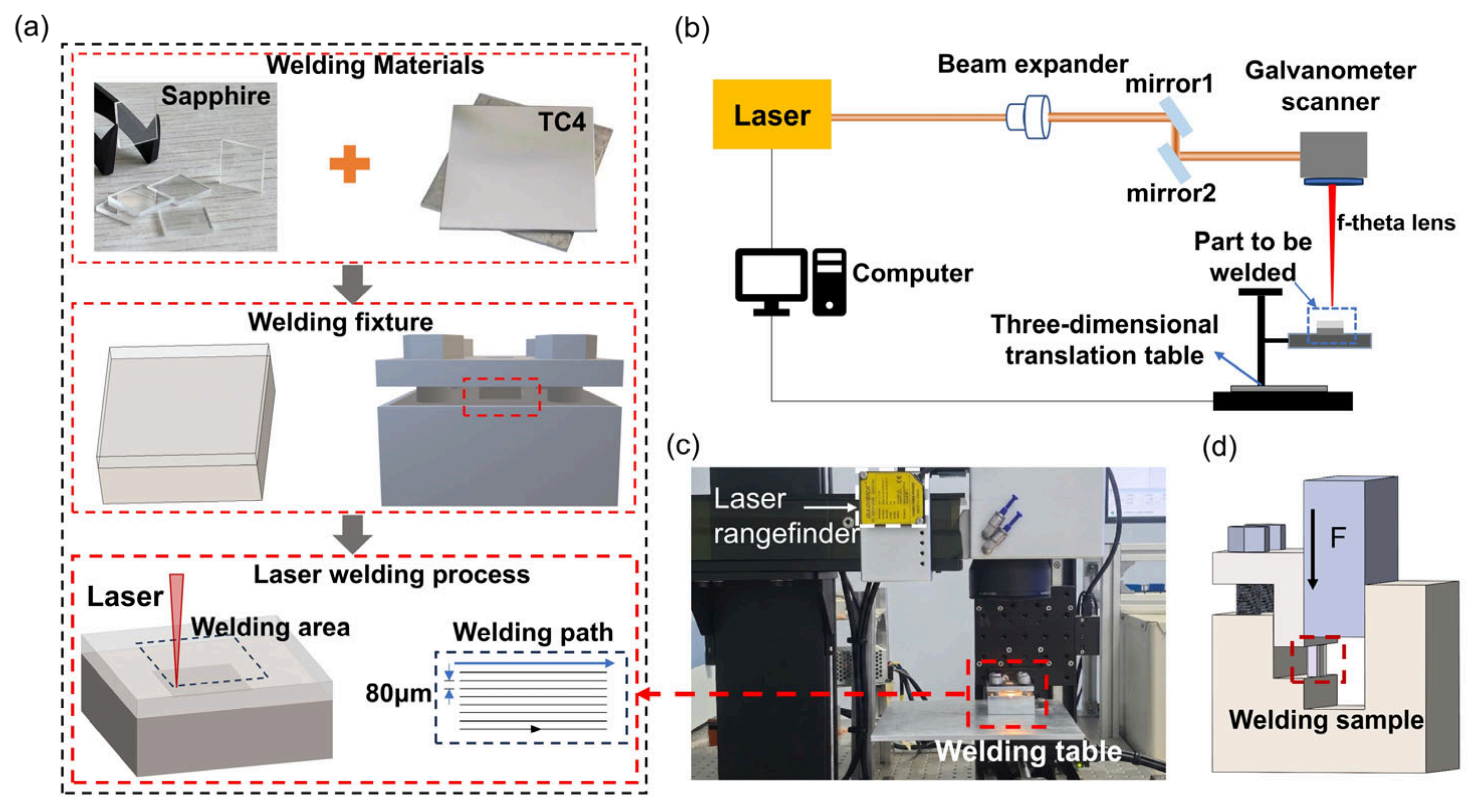
Experimental setup and related apparatus: (**a**) welding process; (**b**) laser system; (**c**) welding platform; and (**d**) shear-testing apparatus.

During welding, the laser was operated in non-burst mode, i.e., as a regular pulse train, at a repetition rate of 800 kHz. The focused spot diameter was approximately 15 μm, and the scanning speed was fixed at 200 mm/s. A line-by-line scanning strategy was adopted to produce a square welded region measuring 6 mm × 6 mm at the interface, with a hatch spacing of 80 μm. Based on preliminary process exploration, three laser powers of 8.4, 12.4, and 16.6 W were selected to systematically investigate the effect of heat input on interfacial reactions and joint properties. These powers cover a complete window ranging from the stage of just forming effective bonding to that of excessive heat input. The experimental setup and related apparatus are shown in Figure 1. The detailed processing parameters are listed in Table 2.

### 2.3. Microstructural Characterization and Mechanical Testing

After welding, the specimens used for the main cross-sectional observations were sectioned perpendicular to the weld direction using a diamond wire-cutting machine. The sectioned specimens were cold-mounted to avoid the influence of hot mounting on the residual stress on the ceramic side and were subsequently subjected to grinding and polishing using an automatic grinding and polishing machine. To evaluate whether the observed interfacial microcracks could have been introduced during metallographic preparation, additional comparative specimens were prepared. For these comparative specimens, the welds were positioned close to the specimen edge so that the relevant cross-sections could be prepared without the diamond wire-cutting step, followed by direct cold mounting, grinding, and polishing. Specimens were also processed at different polishing rotation speeds. No distinct morphological differences or additional cracking were observed among these comparative groups, supporting the interpretation that the observed interfacial microcracks were not primarily introduced by the metallographic preparation procedures.

For each laser-power condition, 2–3 independently welded specimens were examined, and 1–2 representative cross-sections were prepared and examined for each specimen. The cross-sectional SEM observations were used to qualitatively assess the morphology and spatial distribution of interfacial microcracks. Representative fields were selected from these cross-sectional observations for subsequent microstructural analysis. Because the purpose of the cross-sectional SEM examination was qualitative morphological assessment rather than a predefined quantitative crack survey, the number of SEM fields per cross-section was not fixed a priori. Accordingly, the microcracking was evaluated qualitatively rather than using a normalized crack-length metric. The macroscopic morphology and three-dimensional surface morphology of the welds were examined using a laser scanning confocal microscope (LSCM, OLS5100, Olympus, Tokyo, Japan). The interfacial microstructure and fracture-surface morphology of the joints were observed using a field-emission scanning electron microscope (FE-SEM, GeminiSEM 360, ZEISS, Oberkochen, Germany), and the interfacial elemental distributions were analyzed using an energy-dispersive spectrometer (Ultim Max 65 SDD, Oxford Instruments, Abingdon, UK). X-ray diffraction (XRD) analysis was performed on the sapphire-side fracture surfaces using an X-ray diffractometer (DX-2700BH, Dandong Haoyuan Instrument Co., Ltd., Dandong, China) with Cu Kα radiation (λ = 1.5406 Å) in a θ–θ geometry. The X-ray tube was operated at 40 kV and 30 mA. The diffraction patterns were collected in continuous-scan mode over a 2θ range of 10–90°, with a data interval of 0.02° and a scanning rate of 5°/min.

The apparent shear strengths of the joints were measured using a universal testing machine (DWD-100, Jinan Henglexingke Instrument Co., Ltd., Jinan, China). For the shear specimens, the bonding region was positioned slightly offset from the specimen center. During testing, the protruding sapphire edge was loaded directly by the indenter, while the TC4 alloy was firmly clamped using a custom-made fixture. A quasi-static load was applied nominally parallel to the bonding interface at a constant crosshead speed of 0.1 mm/min. Because the load line was offset from the bond plane, a bending component could not be completely eliminated. Therefore, the measured strength is reported as the apparent shear strength.

Fourteen specimens were prepared for each laser-power condition. Among these, nine specimens were subjected to shear testing at 8.4 and 12.4 W, and eight specimens at 16.6 W; the remaining specimens were used for other characterization or retained as backups. A test was excluded from the strength analysis when the load–time curve exhibited an abnormal multi-stage response, characterized by an initial increase in load followed by a decrease and a subsequent increase, or when post-test inspection revealed severe sapphire fragmentation such that the joint-failure load could not be unambiguously identified. Accordingly, two tests were excluded at 8.4 W, two at 12.4 W, and one at 16.6 W, leaving seven valid specimens (*n* = 7) for each condition. All seven valid results were included in the statistical analysis.

The apparent shear strength is reported as the mean ± standard deviation (SD). Statistical differences among the three laser-power conditions were evaluated using one-way analysis of variance (ANOVA), followed by Tukey’s HSD test for pairwise comparisons, with *p* < 0.05 considered statistically significant. The individual apparent shear-strength values are provided in Table S1 of the Supplementary Material. After the shear tests, the fracture-surface morphologies on both the sapphire and TC4 alloy sides were systematically examined by SEM to clarify the failure behavior and fracture mechanism of the joints.

## 3. Results and Discussion

### 3.1. Weld Characteristics

**Figure 2:**
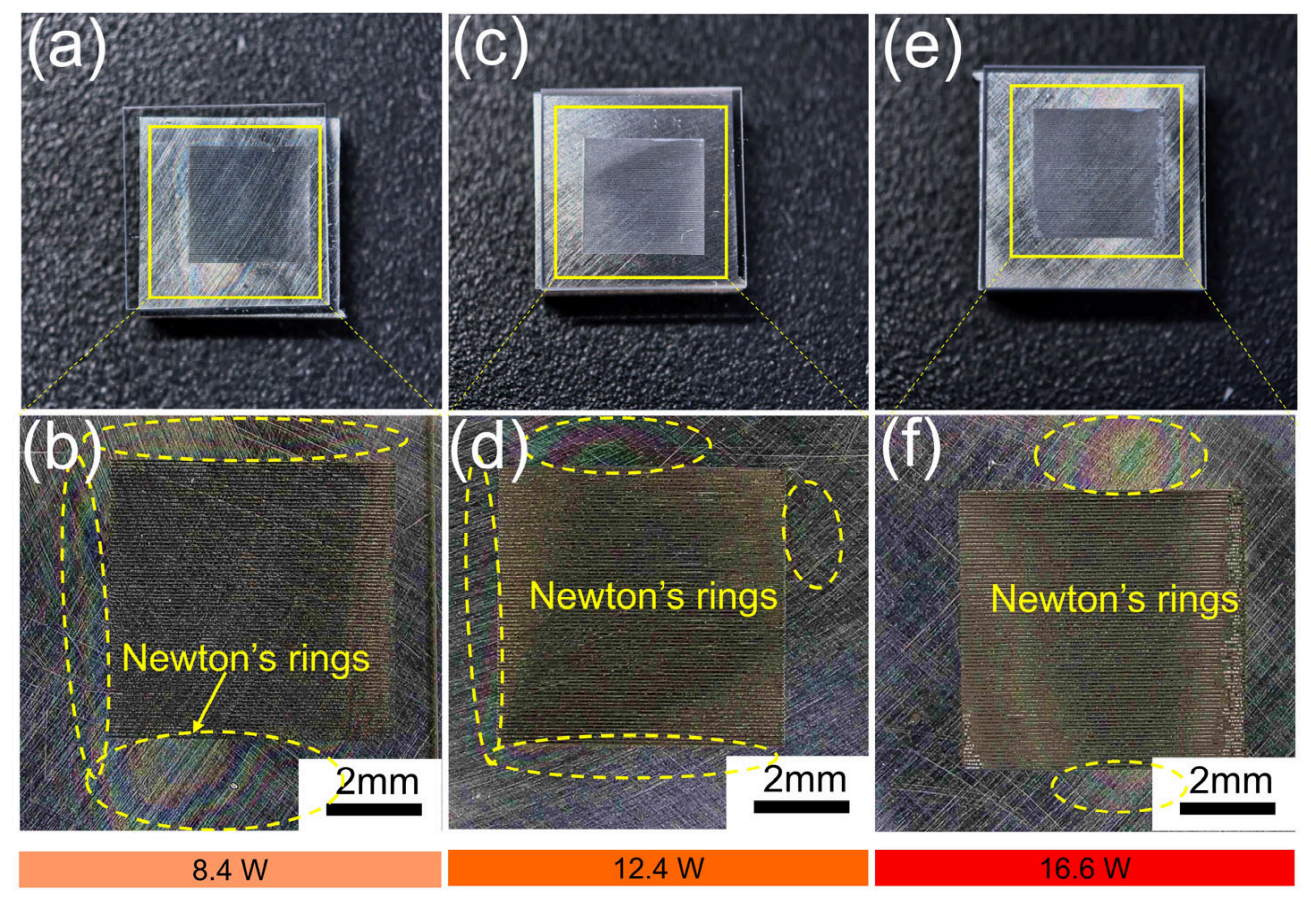
Macroscopic surface morphologies of sapphire/TC4 alloy joints at different laser powers: (**a**,**b**) 8.4 W; (**c**,**d**) 12.4 W; (**e**,**f**) 16.6 W.

The macroscopic morphologies of the sapphire/TC4 alloy joints produced at different average laser powers are shown in Figure 2. Within the investigated power range, continuous and intact bonded regions were formed, and no obvious discontinuities, spatter, surface-visible macroscopic cracks, or through-thickness cracks were observed. Sapphire displayed high optical transparency, and the underlying TC4 alloy substrate remained visible after welding, indicating that the laser interaction did not visibly impair the overall transparency of the sapphire. Colored interference fringes corresponding to Newton’s rings were observed in the unwelded regions of all specimens, whereas such fringes were absent within the welded zones. In general, Newton’s rings originate from optical interference induced by the residual thin air layer between the contacting sapphire and TC4 alloy surfaces. Their continuous distribution requires the interfacial gap to remain continuous and on the nanometer scale [36]. The distribution of Newton’s rings revealed that minor interfacial gaps persisted in unwelded areas. These local gaps were eliminated within welded zones via molten material infiltration and subsequent solidification bonding [37]. These observations demonstrate that, at a high scanning speed of 200 mm/s, femtosecond laser welding can join sapphire and TC4 alloy while preserving the integrity and transparency of the sapphire substrate.

**Figure 3:**
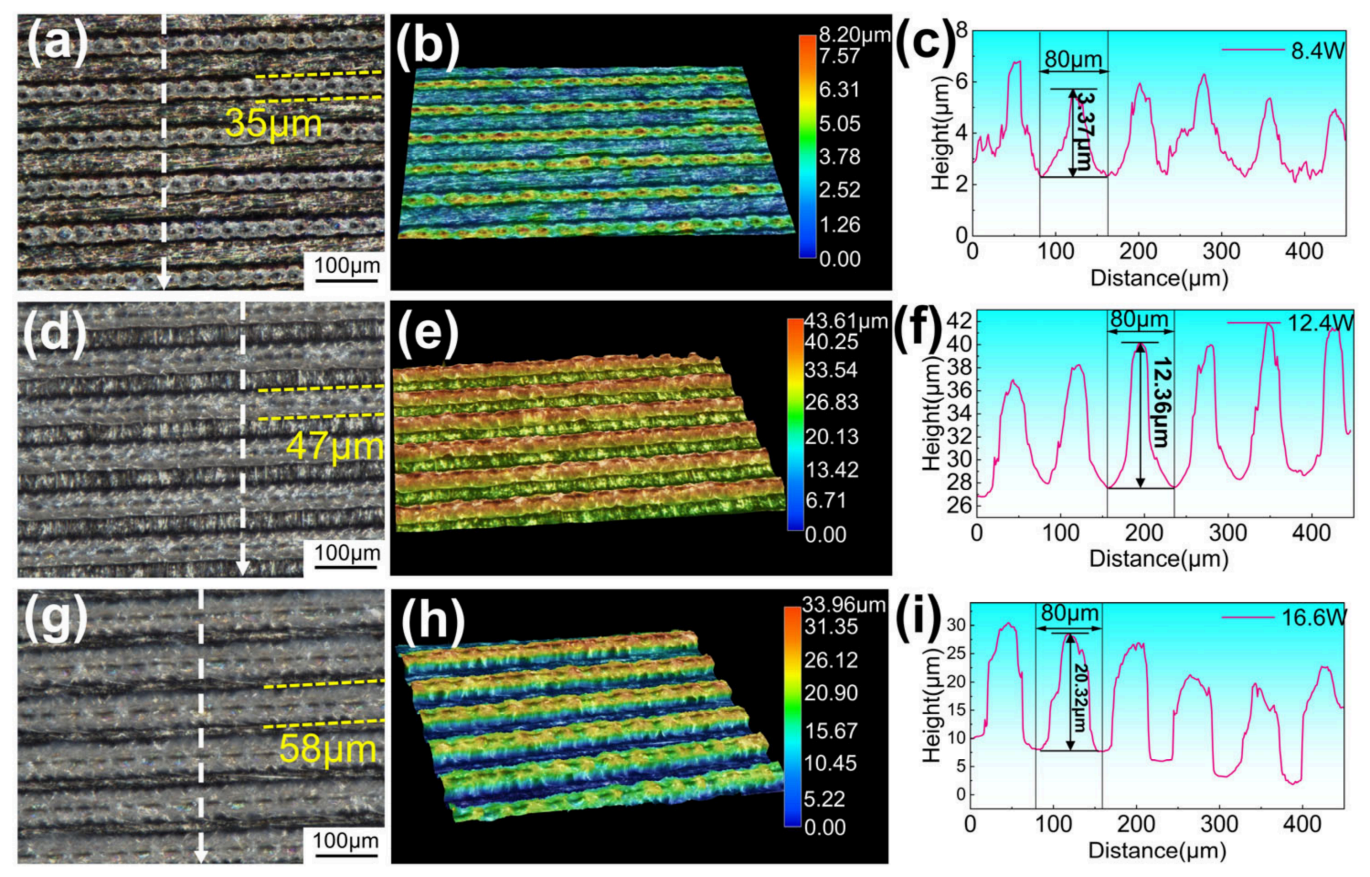
Surface morphologies and related data of sapphire/TC4 joints at different laser powers: (**a**–**c**) 8.4 W; (**d**–**f**) 12.4 W; (**g**–**i**) 16.6 W.

The morphologies of the welded regions produced at different laser powers are shown in Figure 3. Figure 3a,d,g show the planar morphologies, whereas Figure 3b,e,h show the corresponding three-dimensional morphologies. The welded regions exhibited continuous undulating morphologies without obvious collapse or discontinuous defects at the different laser powers. As the laser power increased from 8.4 to 16.6 W, the apparent width of the laser-affected region observed through the sapphire increased from approximately 35 to 58 μm. This optically observed width was used to characterize the variation in the lateral extent of the laser-affected region with increasing laser power. Because the observation was made through the sapphire, the apparent boundary may be affected by the optical response of the laser-modified interfacial region; therefore, this width does not represent the effective bonded-track width used for the mechanical-property calculation. Figure 3c,f,i show the two-dimensional interfacial profile curves extracted from the reconstructed three-dimensional surface morphologies. The interfacial relief height also increased with increasing laser power. At a laser power of 8.4 W, the bonding interface between sapphire and TC4 alloy exhibited a regular, periodically undulating sawtooth-like structure, with a peak height of approximately 3.37 μm. When the laser power was increased to 12.4 W, the interface retained its overall periodic undulation; however, the sharp and distinct sawtooth profile observed at the lower power was markedly weakened. The crests of the undulations became smoother, while the peak height increased to approximately 12.36 μm. When the laser power was further increased to 16.6 W, the local interfacial peak height increased to approximately 20.32 μm, and the lateral dimensions of the individual crests also increased significantly. The interfacial profile thus exhibited a progressive transition from the sharp, angular sawtooth-like undulations observed at low power to a periodic, approximately square-wave-like morphology with more pronounced plateau structures. These observations indicate that increasing laser power promoted more extensive localized melting in the interfacial region. Driven by the combined effects of laser-induced pressure and interfacial confinement, the molten material spread along the sapphire/TC4 interface, producing a more pronounced wavy interfacial morphology. The increased interfacial tortuosity provided a more complex geometrical interface for subsequent interfacial reactions and crack propagation.

### 3.2. Microstructure of Sapphire/TC4 Alloy Joints

**Figure 4:**
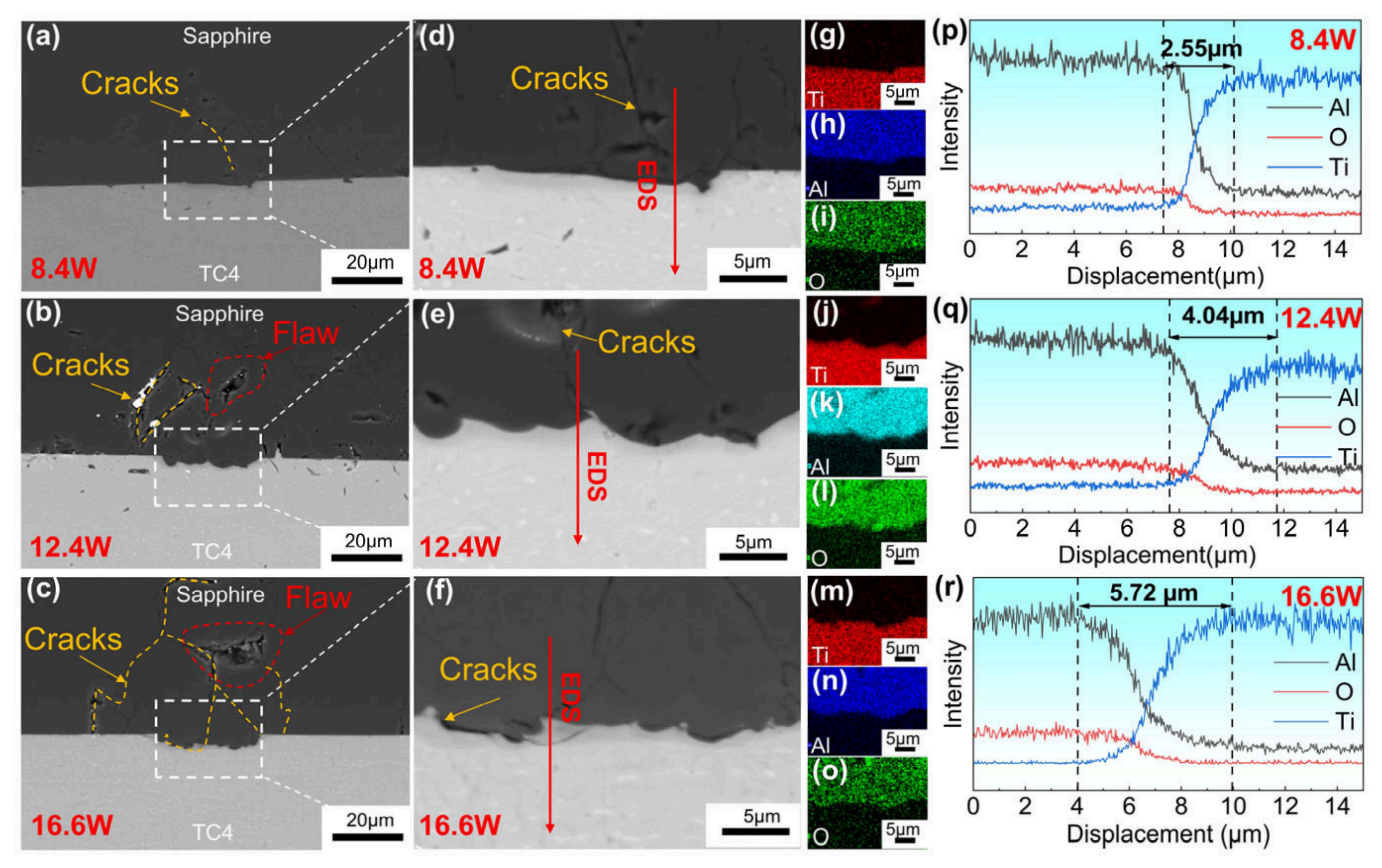
Representative cross-sectional microstructures and elemental distributions of sapphire/TC4 alloy joints at different laser powers: (**a**–**f**) microstructures; (**g**–**r**) elemental distributions.

The microstructure and elemental distributions in sapphire/TC4 alloy joints at different laser powers are shown in Figure 4. As shown in Figure 4a–f, continuous welded regions were formed in all joints, whereas the interfacial morphology and integrity varied markedly with laser power. At 8.4 W, the sapphire/TC4 alloy interface maintained continuous contact and exhibited a relatively flat morphology. Slight localized melting traces were observed on the TC4 alloy side, while only isolated microcracks were observed on the sapphire side. When the laser power was increased to 12.4 W, the sapphire/TC4 alloy interface exhibited a distinct wavy morphology, and a relatively continuous molten filling region formed on the TC4 alloy side. More extensive microcracking was observed in the sapphire adjacent to the interface. When the laser power was further increased to 16.6 W, the lateral dimensions of the wavy interfacial morphology increased markedly, and the molten region on the TC4 alloy side continued to expand. Cracking on the sapphire side became more pronounced, with locally interconnected cracks and partial material loss near the interface. Across the examined cross-sections, the observed microcracks were localized within the laser-scanned tracks, whereas the regions between adjacent tracks remained intact. This spatial correspondence strongly supports a welding-related origin of the observed cracking.

The elemental maps (Figure 4g–o) show overlapping distributions of Ti, Al, and O at the sapphire/TC4 alloy interface at different laser powers. This indicates that the sapphire/TC4 alloy interface exceeded mere physical contact under femtosecond laser irradiation, with elemental diffusion occurring across the interface. To further characterize the distributions of the constituent elements, EDS line-scan analyses were performed across the interface (Figure 4p–r). It can be seen that the width of the interfacial compositional transition zone gradually increased from approximately 2.55 μm to 5.72 μm as the laser power increased from 8.4 to 16.6 W. At 8.4 W, the Ti, Al, and O signals changed relatively sharply across the interface, indicating limited elemental diffusion. At 12.4 W, the extension of the Ti distribution toward the sapphire side became more pronounced, while Al and O exhibited relatively continuous transition profiles on the TC4 alloy side. These profiles indicated more pronounced elemental intermixing across the sapphire/TC4 alloy interface compared with the joint produced at 8.4 W. When the power was increased to 16.6 W, the elemental concentration gradients became more gradual, and the transition zone reached its maximum width, indicating that the higher heat input promoted more extensive elemental migration across the interface. It should be noted that a distinct interfacial reaction layer could not be directly resolved in the present secondary-electron images; therefore, its thickness was not determined by SEM/EDS. The 2.55–5.72 μm values obtained from the EDS line scans in Figure 4 represent the width of the interfacial compositional transition zone, reflecting the extent of elemental diffusion and intermixing, rather than a directly measured reaction-layer thickness. XRD identified the interfacial reaction products, while EDS elemental-distribution analysis revealed redistribution and intermixing of Ti, Al, and O across the interface.

**Figure 5:**
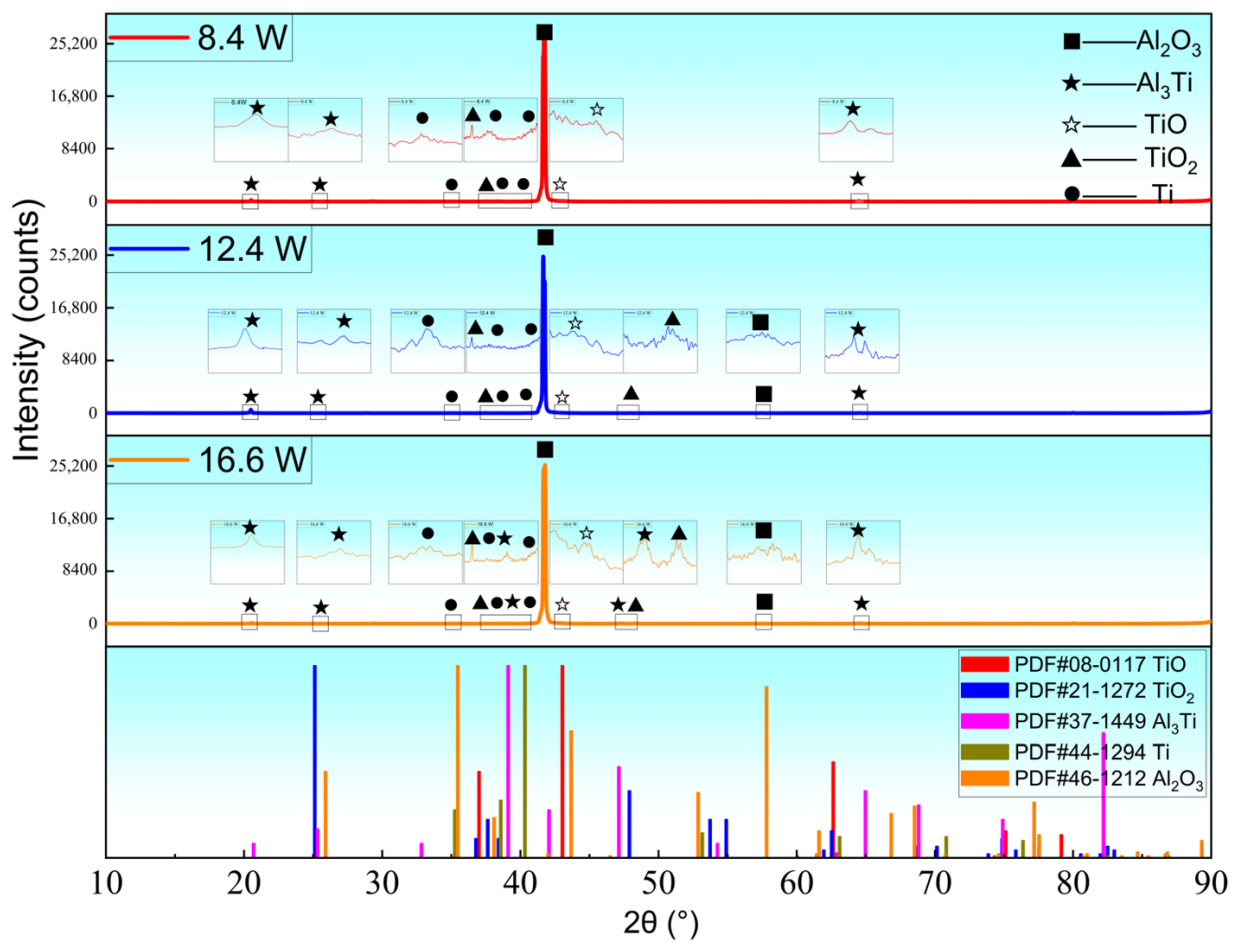
XRD patterns of the sapphire-side fracture surfaces at different laser powers, with enlarged weak-reflection regions and corresponding PDF/ICDD reference peak positions.

Figure 5 shows the XRD patterns obtained from the sapphire-side fracture surfaces of the joints. All three representative patterns are plotted on the same intensity scale. Because the α-Al_2_O_3_ reflections are substantially stronger than the weak reflections associated with the interfacial reaction products, selected angular regions are additionally shown as enlarged insets of the corresponding experimental data. The experimental patterns were compared with the reference peak positions from the corresponding PDF/ICDD cards, which are overlaid in Figure 5. In addition to the dominant α-Al_2_O_3_ reflections, diffraction features attributable to TiO, TiO_2_, and Al_3_Ti were identified, supporting the occurrence of Ti–O and Ti–Al metallurgical reactions at the interface.

The assignment of α-Ti was also re-examined using accompanying reflections rather than the ~38° reflection alone. The reference positions of the α-Ti (100), (002), and (101) reflections near 35.1°, 38.4°, and 40.2°, respectively, are indicated in Figure 5. The Al_3_Ti (112) reference position near 39° is also included to account for possible overlap in this angular region. Thus, the phase assignments in the 35–41° region were made by comparison with multiple reference reflections rather than on the basis of a single diffraction peak. Together with the EDS line-scan results, which showed that the width of the interfacial compositional transition zone increased from 2.55 μm to 5.72 μm with increasing laser power, the XRD results further support the occurrence of interfacial elemental redistribution and metallurgical reactions. This trend is consistent with previous ultrafast-laser joining studies showing that increased energy input can promote interfacial elemental migration and reaction-product formation [24].

### 3.3. Metallurgical Reactions in the Sapphire/TC4 Alloy Joints

Under the present experimental conditions, the ultrashort pulse duration of 211 fs and the high scanning speed of 200 mm/s resulted in a rapid nonequilibrium thermal cycle at the sapphire/TC4 alloy interface. Previous studies have reported high cooling rates in ultrafast-laser-affected regions [38]. For comparison, nanosecond laser welding has been reported to produce a larger heat-affected zone than ultrashort-pulse laser welding because of its longer pulse–material interaction time [39]. These characteristics indicate that the thermal effect of ultrashort-pulse laser processing is highly localized. Such rapid and localized thermal cycling may have limited the time available for long-range elemental diffusion and the growth of interfacial reaction products. Consequently, the reaction products were likely confined within the narrow interfacial compositional transition zone identified in the present joints rather than forming a distinct thick reaction layer.

Under laser irradiation, localized melting occurred near the sapphire/TC4 alloy interface, and Al, Ti, and O were the primary elements involved in interfacial mixing and redistribution. The coexistence of a transient liquid phase, elemental transport, and interfacial reactions formed a transient reactive metal/ceramic interfacial region in which interfacial reactions and liquid spreading were closely coupled [32,33,34]. To evaluate the thermodynamic driving forces for possible interfacial reaction-product formation, four representative reactions involving Al, Ti, and O were considered, as given in Equations (1)–(4):

Al_2_O_3_ + 3Ti = 2Al + 3TiO (1)

3Al + Ti = Al_3_Ti (2)

Al + 3Ti = Ti_3_Al (3)

2TiO + O_2_ = 2TiO_2_ (4)

Temperature-dependent thermodynamic functions for Al, Ti, Al_2_O_3_, TiO, TiO_2_, O_2_, and Al_3_Ti were obtained from the thermochemical data compiled by Tian and Pang [40], while the thermodynamic data for Ti_3_Al were supplemented according to Witusiewicz et al. [41]. Phase-transition contributions contained in the adopted thermodynamic data were considered within their applicable temperature ranges. The Gibbs free-energy change of each reaction, *ΔG*, was calculated according to the stoichiometry of the corresponding reaction and the adopted reference states.

Because Reactions (1)–(3) consume different stoichiometric amounts of Ti (3, 1, and 3 mol, respectively), their *ΔG* values expressed per mole of reaction cannot be directly compared to evaluate the relative tendencies of the competing Ti-consuming pathways. Since metallic Ti is the common reactant in these reactions, the *ΔG* values were additionally normalized to one mole of Ti consumed to provide a common stoichiometric basis for comparison. For a direct quantitative comparison of Reactions (1)–(3), the temperature range was restricted to 500–1400 K, corresponding to their common applicable range, whose upper limit is constrained by the thermodynamic data available for the intermetallic phases involved in Reactions (2) and (3). This restriction does not imply that either the peak interfacial temperature or the onset of cooling is 1400 K. The actual transient interfacial temperature during laser irradiation can exceed 1400 K. Rather, 1400 K represents the upper boundary below which Reactions (1)–(3) can be directly compared on a common Ti-normalized basis without extrapolating the thermodynamic data of Al_3_Ti and Ti_3_Al beyond their applicable ranges. Representative values at 1400 K and the corresponding reference states are summarized in Table 3.

At 1400 K, the calculated *ΔG* values of Reactions (1)–(4) are all negative, indicating that these candidate reactions are thermodynamically feasible under the adopted reference states. After normalization to one mole of Ti consumed, the *ΔG* values of Reactions (1), (2), and (3) are −0.029, −95.326, and −25.055 kJ·mol^−1^ Ti, respectively. Among these Ti-consuming reactions, Reaction (2), corresponding to Al_3_Ti formation, exhibits the most negative Ti-normalized *ΔG* and therefore the strongest thermodynamic driving force under the adopted reference states.

**Figure 6:**
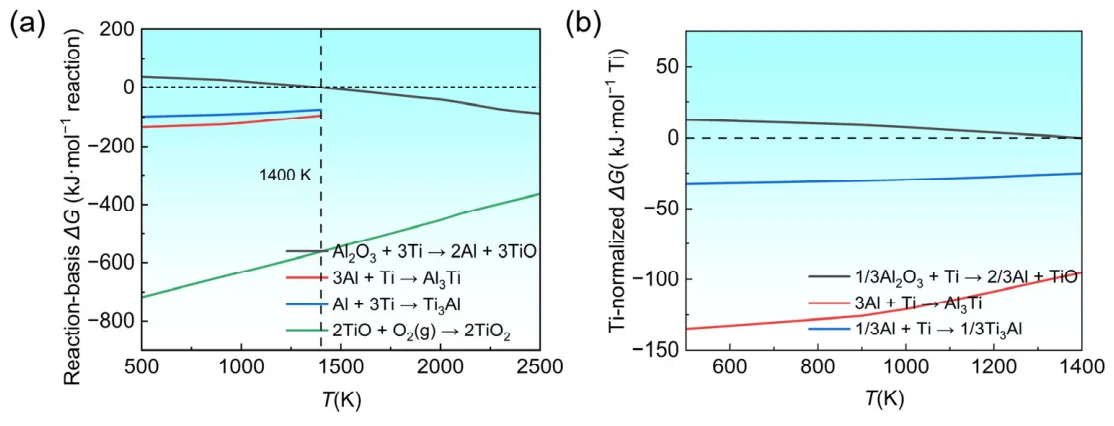
Temperature dependence of the Gibbs free-energy changes of the candidate interfacial reactions: (**a**) reaction-basis *ΔG*; (**b**) Ti-normalized *ΔG* of Reactions (1)–(3).

Figure 6a shows the temperature dependence of the reaction-basis *ΔG* for each candidate reaction within the applicable range of the corresponding thermodynamic data. These curves are used to evaluate the thermodynamic feasibility and temperature dependence of each reaction individually, including the high-temperature region above 1400 K where supported by the corresponding dataset, rather than to directly rank reactions with different stoichiometries. Figure 6b, in contrast, provides a direct comparison of Reactions (1)–(3) on the common Ti-normalized basis over 500–1400 K. At the upper boundary of this common interval, 1400 K, the Ti-normalized *ΔG* of the Ti–Al_2_O_3_ displacement reaction is only slightly negative and close to zero, whereas those of the Al–Ti reactions remain substantially more negative. Within the 500–1400 K interval, further cooling makes the Ti-normalized *ΔG* values of the Al–Ti reactions progressively more negative, while that of the Ti–Al_2_O_3_ displacement reaction increases and becomes thermodynamically less favorable. This divergence indicates that the relative thermodynamic tendency toward Al–Ti intermetallic formation becomes increasingly pronounced as cooling proceeds within the common comparison range, particularly for the Al_3_Ti-forming Reaction (2). Combined with the XRD identification of Al_3_Ti in the welded joints, the thermodynamic results support the formation and retention of Al_3_Ti during the rapid cooling stage. Reaction (4) represents the subsequent oxidation of TiO rather than a competing reaction consuming metallic Ti; its negative *ΔG* indicates that further oxidation of TiO to TiO_2_ is thermodynamically feasible when oxygen is locally available. Overall, the Gibbs free-energy analysis establishes the thermodynamic feasibility of the candidate interfacial reactions and, for Reactions (1)–(3), their relative tendencies on a common Ti-normalized basis under the adopted reference states. In the framework of high-temperature capillarity, reaction-induced changes in local chemistry and reaction-product formation can alter the structure and energetics of the solid–liquid interface, thereby affecting wetting and spreading [32,34,42]. Reactive wetting and spreading can therefore evolve together with liquid transport and interfacial reaction kinetics [43,44]. In the present femtosecond process, these coupled interfacial processes took place during rapid nonequilibrium heating and subsequent solidification.

**Figure 7:**
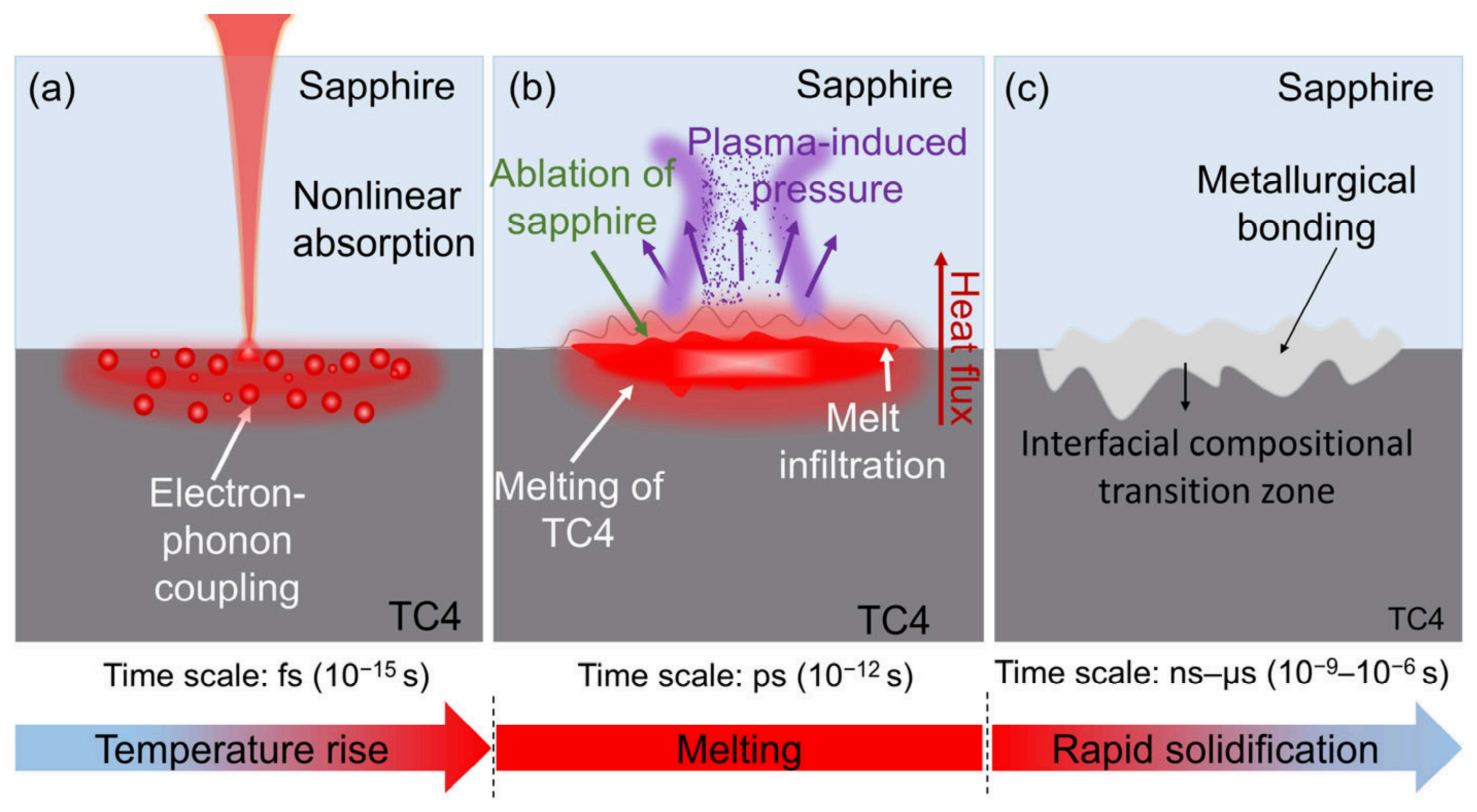
Schematic illustration of the femtosecond-laser-induced joining mechanism at the sapphire/TC4 alloy interface: (**a**) heating stage; (**b**) melting stage; and (**c**) cooling stage.

According to the studies of Wellershoff et al. [45] and Zuo et al. [46], the femtosecond-laser-induced joining mechanism of sapphire/TC4 alloy is primarily governed by nonequilibrium physical-metallurgical processes. As illustrated in Figure 7, during welding, the femtosecond laser was focused at the sapphire/TC4 alloy interface. On the TC4 alloy side (Figure 7a), laser energy was initially absorbed by free electrons within the metal matrix. This energy was quickly transferred to the lattice through electron–phonon coupling, which induced transient heating, localized melting and ablation of the TC4 alloy near the interface, as well as plasma formation and expansion. On the sapphire side (Figure 7b), localized electric fields associated with the interfacial plasma promoted multiphoton and avalanche ionization. This process caused localized breakdown, ablation, and structural modification near the sapphire interface and may have promoted the breaking and rearrangement of Al–O bonds. Subsequently, under the combined effects of plasma recoil pressure and interfacial confinement, the transient molten material spread and filled the micro-ablation regions near the interface. Meanwhile, short-range migration and redistribution of Ti, Al, and O altered the local interfacial composition, while the accompanying interfacial reactions modified the solid–liquid interfacial state and thereby influenced wetting and spreading [34,44]. Thus, pressure-assisted melt motion, reactive wetting and spreading, elemental transport, and bond rearrangement acted cooperatively during interfacial evolution. During subsequent rapid cooling and solidification, Ti–O reaction products were retained. As the interface cooled through the common 500–1400 K comparison range, the calculated Ti-normalized *ΔG* of the Al_3_Ti-forming reaction became progressively more negative, indicating an increasing thermodynamic driving force for Al_3_Ti formation during this stage. The resulting Al_3_Ti could then be retained after rapid solidification, consistent with the XRD results. Together, these coupled interfacial processes and subsequent rapid solidification established the bonded interface. As illustrated in Figure 7c, the resulting reaction products were retained within the interfacial compositional transition zone.

### 3.4. Effect of Laser Power on the Characteristics of Sapphire/TC4 Alloy Joints

**Figure 8:**
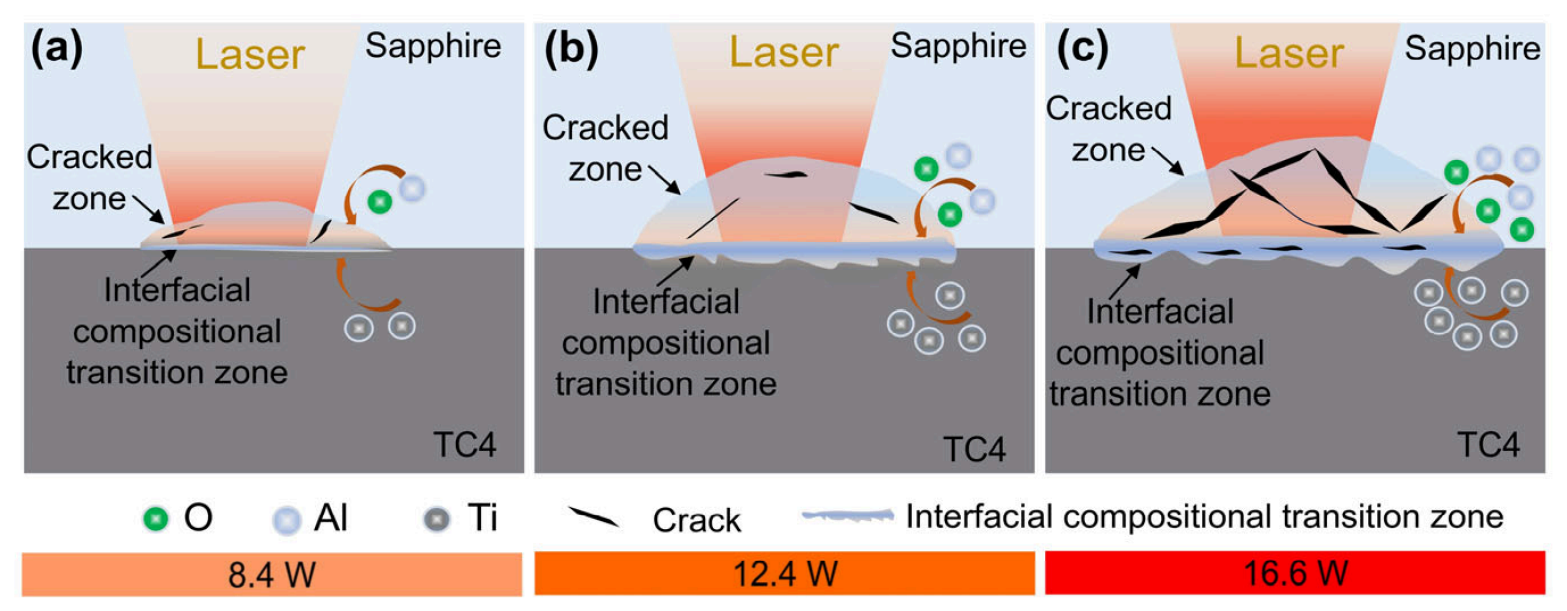
Effect of laser power on the microstructure in sapphire/TC4 alloy joints: (**a**) 8.4 W; (**b**) 12.4 W; and (**c**) 16.6 W.

According to the analytical results presented in Section 3.1, Section 3.2 and Section 3.3, femtosecond laser welding achieved bonding between sapphire and TC4 alloy through instantaneous high-energy deposition. Under laser irradiation, the joint interface experienced transient high-temperature exposure, short-range elemental interdiffusion, interfacial metallurgical reactions, and rapid quenching, producing a nonequilibrium interfacial compositional transition zone containing reaction products. Differences in heat input governed the peak interfacial temperature, the extent of the laser-affected region, the local interfacial melting behavior, the reaction behavior of Ti, Al, and O, and the local thermophysical mismatch. These factors jointly determined the extent of liquid-phase spreading, interfacial morphology, elemental redistribution, and phase assemblage within the transition zone. Figure 8 demonstrates how laser power modulates the interfacial morphology of the welded joints.

As displayed in Figure 8a, at a laser power of 8.4 W, the instantaneous energy deposition was the lowest among the three investigated conditions. Nevertheless, localized interfacial melting occurred on both sides of the directly irradiated sapphire/TC4 interface, indicating that the transient temperature in the focal region was sufficiently high to reach the melting regime of both materials. Sapphire has a melting point of approximately 2050 °C [47], whereas TC4 alloy has a melting range of approximately 1604–1660 °C [48]. Therefore, the main characteristic of the 8.4 W condition was not an insufficient local peak temperature, but rather the limited spatial extent of the high-temperature and molten regions. Because heat was rapidly dissipated into the surrounding base materials, the molten zone remained narrow, thereby limiting the lateral extent of liquid-phase spreading along the interface. Consequently, the sapphire/TC4 alloy bonding interface maintained an overall relatively flat profile, and the extent of elemental redistribution remained comparatively limited. During subsequent rapid cooling, localized thermal stresses developed near the interface, and isolated microcracks were observed on the sapphire side.

As presented in Figure 8b, the heat input from the transient heat source increased remarkably when the laser power was elevated to 12.4 W. Interfacial constraint coupled with laser-induced transient pressure drove further expansion of the localized melting region on the TC4 alloy side. The increased heat input was expected to increase both the local peak temperature and the spatial extent of the molten region. The enlarged molten region and improved fluidity enabled more extensive liquid spreading along the sapphire/TC4 interface. Concurrently, enhanced elemental redistribution and interfacial reactions accompanied the more extensive liquid spreading and modified the local solid–liquid interfacial state. As a result, the bonding interface evolved from the less pronounced interfacial undulation observed at low laser power into a well-defined periodic wavy morphology. Elevated temperatures further facilitated short-range interdiffusion of Ti, Al, and O and promoted interfacial metallurgical reactions. Meanwhile, high heat input aggravated the accumulation of interfacial thermal stress. More extensive microcracking was observed on the sapphire side than under the 8.4 W condition.

As presented in Figure 8c, the laser power was further increased to 16.6 W, accompanied by continuously rising transient heat input. The molten zone on the TC4 alloy side expanded further, thereby increasing the spreading range of the liquid phase. Accordingly, the periodic width of the wavy interfacial structure increased remarkably. Individual undulations also exhibited an evident rise in lateral size. The elevated heat input widened the molten pool and further intensified interfacial metallurgical reactions. At the same time, thermal stress accumulated continuously on the sapphire side and aggravated stress damage. Initially isolated microcracks interconnected and propagated to form continuous crack bands. The reaction-product-containing interfacial region also exhibited limited ability to accommodate shrinkage deformation during cooling [49]. Excessive transient thermal loading induced severe stress concentration at the bonding interface. When the local stress exceeded the fracture resistance of the reaction-product-containing interfacial region, microcracks initiated and propagated along the interface. These observations suggest that excessive thermal loading and the associated thermal stresses promoted crack initiation and propagation at the sapphire/TC4 alloy interface under the 16.6 W condition.

To further assess the contribution of thermal-expansion mismatch to crack formation, a simplified order-of-magnitude estimate was made. Representative thermal expansion coefficients of sapphire and TC4 alloy were taken as approximately 7.5 × 10^−6^ K^−1^ and 8.6 × 10^−6^ K^−1^, respectively [50,51], giving a thermal-expansion mismatch of approximately 1.1 × 10^−6^ K^−1^. Because thermal-mismatch stress can be mechanically sustained mainly after the locally molten interfacial region has resolidified, the solidus temperature of TC4 alloy, approximately 1604 °C [48], was taken as a simplified reference temperature for the onset of solid-state constraint. Assuming cooling from 1604 °C to room temperature (25 °C), *ΔT* was approximately 1579 K, corresponding to a thermal-mismatch strain of approximately 1.74 × 10^−3^. For a simple elastic order-of-magnitude estimate, the characteristic thermal-mismatch stress scale was approximated as *σ* ≈ *E* × *Δα* × *ΔT*. Using an in-plane Young’s modulus of approximately 431 GPa for C-plane sapphire [52] gives a stress scale of approximately 0.75 GPa. This value should be regarded as an order-of-magnitude estimate rather than the actual residual stress, because the local stress state is additionally affected by temperature-dependent material properties, plastic relaxation of TC4 alloy, local melting and solidification, interfacial geometry, and stress relaxation associated with cracking. Nevertheless, the estimate indicates that the thermal-expansion mismatch can generate substantial local stresses during rapid cooling, consistent with the microcracking observed in the laser-scanned regions.

### 3.5. Mechanical Properties and Fracture Behavior of Sapphire/TC4 Alloy Joints

**Figure 9:**
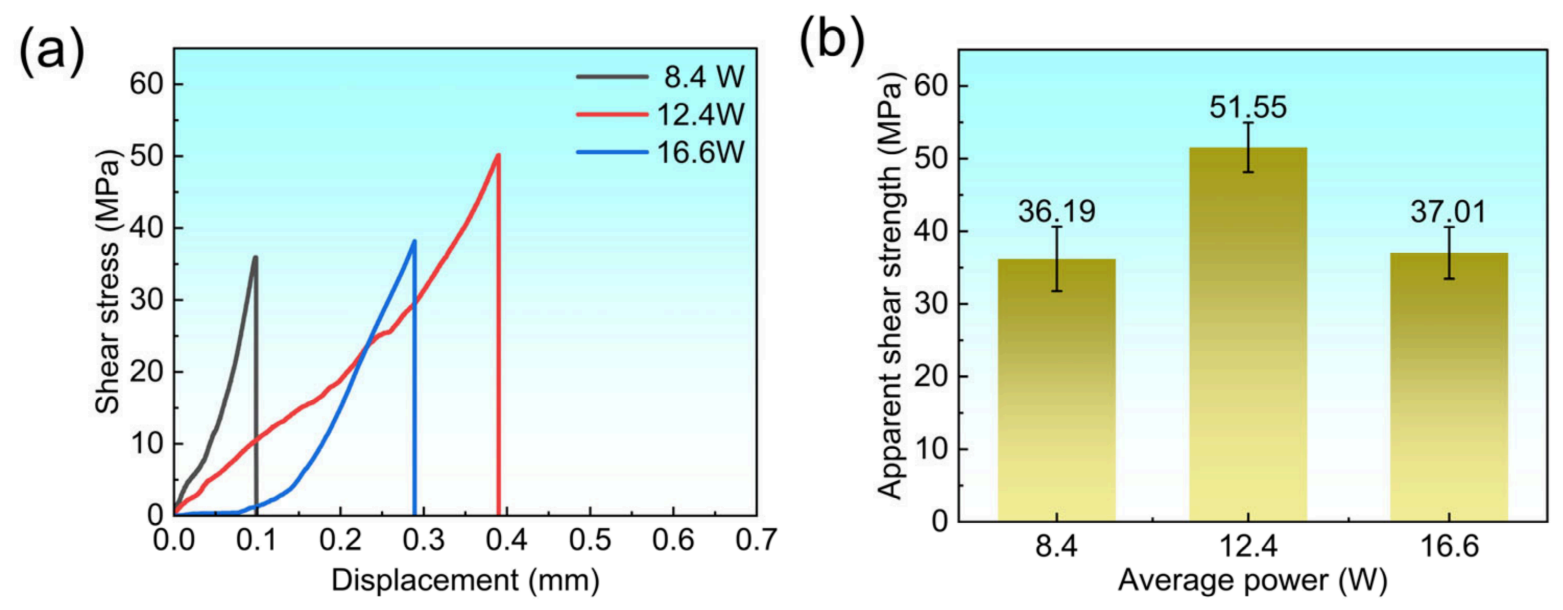
Mechanical properties of sapphire/TC4 alloy joints at different laser powers: (**a**) representative shear stress–displacement curves; (**b**) apparent shear strength. Error bars represent SD (*n* = 7).

**Figure 10:**
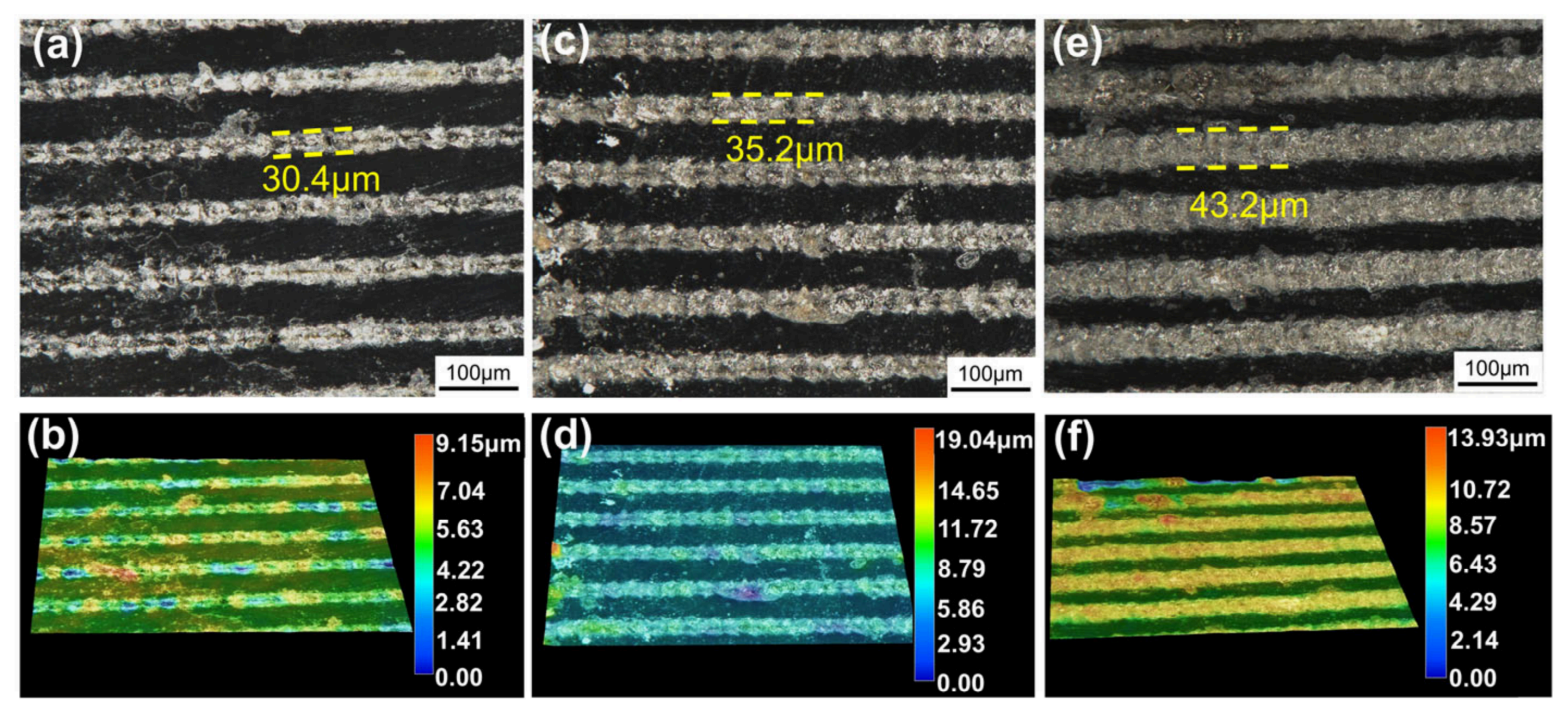
Morphologies of the TC4 alloy side after shear testing at different laser powers: (**a**,**b**) 8.4 W; (**c**,**d**) 12.4 W; and (**e**,**f**) 16.6 W. The annotated values indicate the effective bonded-track widths.

The apparent shear-strength test results of the welded joints are shown in Figure 9. The shear stress shown in Figure 9a was calculated as an apparent stress according to Equation (5) [53]:

*τ* = *F*/*S* (5)

where *τ* is the apparent shear stress (MPa), *F* is the applied load (N), and *S* is the estimated effective bonded area (mm^2^). The peak value of *τ* was taken as the apparent shear strength of the joint. The nominal scanned region was 6 × 6 mm^2^ (*A*_0_ = 36 mm^2^), with a hatch spacing *h* of 80 μm. Because the effective bonded-track widths were smaller than the hatch spacing, adjacent bonded tracks did not overlap. The effective bonded area was therefore estimated as *S* = *A*_0_ (*w*/*h*), where *w* is the effective bonded-track width measured from the post-shear TC4-side fracture surface. The measured values of *w* were approximately 30.4, 35.2, and 43.2 μm at 8.4, 12.4, and 16.6 W, respectively, as shown in Figure 10, giving effective bonded areas of 13.68, 15.84, and 19.44 mm^2^. These values correspond to approximately 38%, 44%, and 54% of the nominal 36 mm^2^ scanned area, respectively. The bonded-track widths used here are distinct from the apparent laser-affected widths observed optically through the sapphire in Section 3.1. Because the apparent shear strength was calculated using the corresponding effective bonded area for each condition, the variation in bonded-track width with laser power was already accounted for in the normalization and was therefore not used to explain the differences in apparent shear strength.

Figure 9a presents representative shear stress–displacement curves selected from the seven valid tests for each laser-power condition. The displacement was calculated from the recorded loading time using the actual crosshead speed of 0.1 mm/min. Because the measured displacement includes compliance contributions from the specimen, fixture, and testing machine, it is not regarded as the local deformation of the welded joint, and the curve slopes are therefore not interpreted as the stiffness of the welded joint.

As shown in Figure 9b, the mean apparent shear strengths at 8.4, 12.4, and 16.6 W were 36.19 ± 4.78, 51.55 ± 3.69, and 37.01 ± 3.84 MPa, respectively (mean ± SD, *n* = 7). One-way ANOVA indicated a significant effect of laser power on the apparent shear strength (*F*(2, 18) = 30.60, *p* < 0.001). Tukey’s HSD test showed that the apparent shear strength at 12.4 W was significantly higher than those at 8.4 and 16.6 W (both *p* < 0.001), whereas no significant difference was found between 8.4 and 16.6 W (*p* = 0.927), indicating that their apparent shear strengths were comparable within the experimental scatter. Despite their comparable apparent shear strengths, the fracture and microstructural observations discussed below reveal different limiting mechanisms under these two conditions.

The fracture morphologies produced at different laser powers were systematically characterized. Figure 10 shows the three-dimensional depth-of-field morphologies of the shear-fractured surfaces on the TC4 alloy side. As the laser power increased from 8.4 to 16.6 W, the surface undulation within the laser-affected region on the metal side generally increased. Based on the surface-height distributions, the characteristic undulation height increased from approximately 9.15 μm at 8.4 W to 11.72 μm at 12.4 W and 13.93 μm at 16.6 W. At 12.4 W, only isolated local peaks approached 19.04 μm, whereas most of the elevated region was concentrated near 11.72 μm. These results indicate that increasing laser power generally enlarged the laser-affected region and enhanced the melting response on the TC4 alloy side. In addition, the widths measured from the post-shear TC4-side fracture surfaces were approximately 30.4, 35.2, and 43.2 μm at 8.4, 12.4, and 16.6 W, respectively. These fracture-surface widths were taken as the effective bonded-track widths for estimating the effective bonded areas used in Equation (5). They are distinct from the apparent laser-affected widths of 35–58 μm observed optically through the sapphire in Section 3.1.

**Figure 11:**
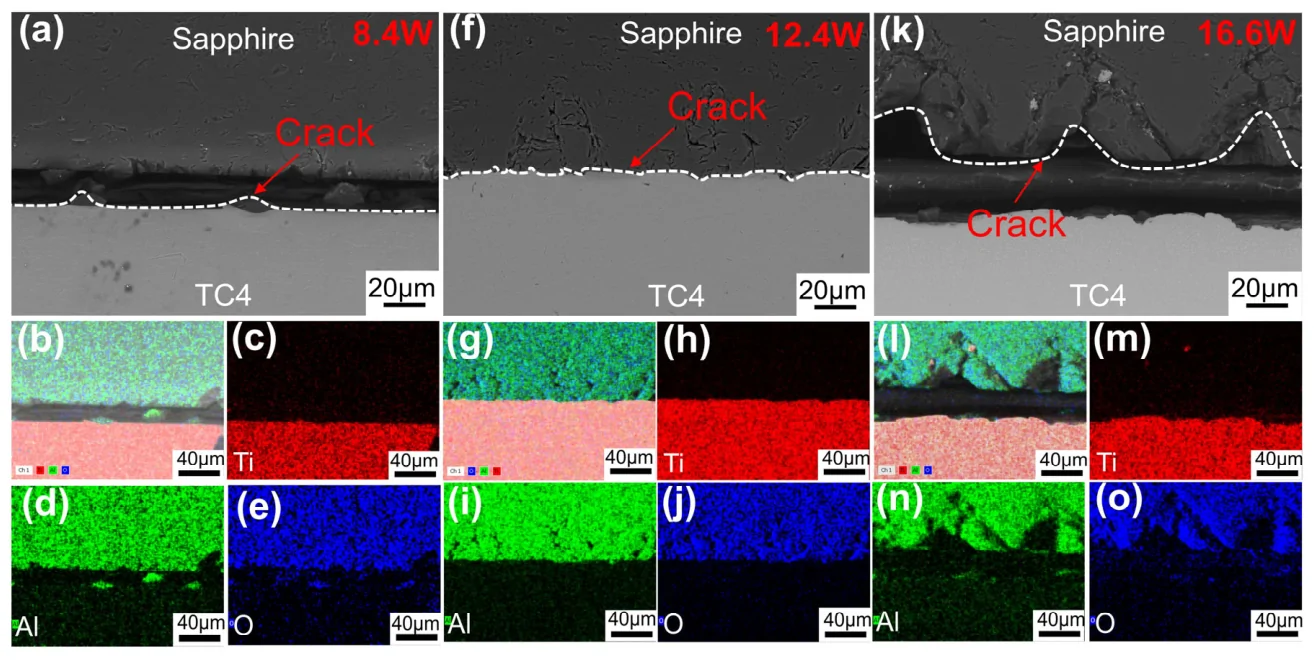
Fracture paths and elemental distributions in the joint cross-sections after shear failure at different laser powers: (**a**–**e**) 8.4 W; (**f**–**j**) 12.4 W; and (**k**–**o**) 16.6 W.

Figure 11 shows the fracture paths of sapphire/TC4 alloy joints and the corresponding element distributions. Under the 8.4 W condition (Figure 11a–e), the main crack propagated almost linearly along the sapphire/TC4 alloy interface, and the crack edges were relatively smooth. Ti, Al, and O exhibited clearly separated distributions on the two sides of the fracture edge, with changes in the crack propagation direction occurring only in localized regions. When the laser power was increased to 12.4 W (Figure 11f–j), the crack propagation path exhibited multiple pronounced deflections. Combined with the interfacial microstructures shown in Figure 4, this phenomenon was closely related to the change in interfacial morphology. Increasing the laser power promoted more extensive interfacial melting, elemental redistribution, and metallurgical reactions, transforming the bonding interface from a relatively flat cross-sectional morphology into a pronounced wavy morphology. During shear loading, this morphology readily generated a mechanical interlocking effect, reduced the stress at the crack tip, and hindered crack propagation, thereby contributing to the higher apparent shear strength at 12.4 W. Under the 16.6 W condition (Figure 4 and Figure 11k–o), the crack extended more prominently into the sapphire substrate during propagation. Although this fracture path appeared to indicate stronger mechanical interlocking, the analysis of Figure 4 showed that numerous continuously distributed microcracks were present on the sapphire side under this power condition, accompanied by material loss in the metallurgically affected region on the sapphire side. These defects provided low-resistance paths for crack propagation and therefore weakened the strengthening effect of the mechanical interlocking structure. This indicates that the reaction-product-containing interfacial region, together with the residual thermal stresses generated by excessive heat input, provided preferential crack-initiation sites near the interface. Although the crack path was geometrically more complex under this condition, these cracks did not constitute beneficial crack-deflection and crack-arrest structures. Instead, they formed damage paths associated with thermal stress and interfacial damage. Under external loading, these cracks readily interconnected and propagated rapidly, ultimately causing unstable fracture of the joint and reducing its strength.

**Figure 12:**
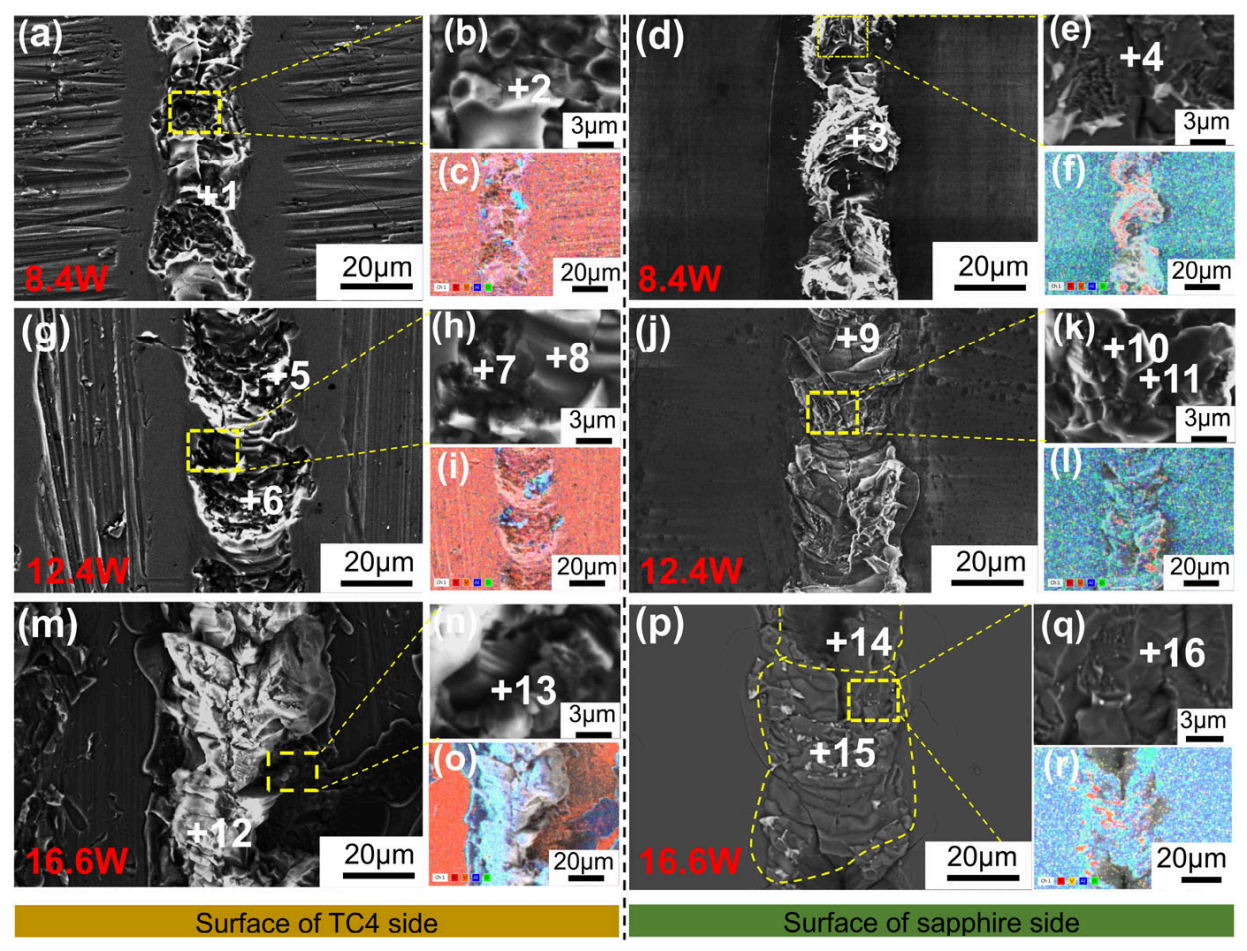
Fracture surface microstructure and elemental distributions of sapphire/TC4 alloy joints at different laser powers. TC4 alloy side: (**a**–**c**) 8.4 W, (**g**–**i**) 12.4 W, and (**m**–**o**) 16.6 W; sapphire side: (**d**–**f**) 8.4 W, (**j**–**l**) 12.4 W, and (**p**–**r**) 16.6 W.

Figure 12 presents the fracture-surface morphologies and elemental distributions on the sapphire and TC4 alloy sides of the joints produced at different laser powers. The chemical compositions of the marked positions are summarized in Table 4, in which all values are given in atomic percent (at%). For the locations assigned to sapphire-rich fracture regions (Points 1, 3, 5, 12, 14, 15, and 16), the measured Al:O atomic ratios range from approximately 1:1.30 to 1:1.93, whereas the stoichiometric Al:O ratio of α-Al_2_O_3_ is 1:1.50. The spread of the measured ratios corresponds to deviations of up to approximately 30% from the stoichiometric value, indicating appreciable uncertainty in EDS oxygen quantification under the present measurement conditions. Therefore, the EDS-derived oxygen contents and Al:O ratios are treated as semi-quantitative compositional information and are not used as exact stoichiometric criteria for phase identification.

Figure 12a–f show the fracture microstructure and EDS results of joints prepared at a laser power of 8.4 W, and Figure 12b,e is magnified views of local regions. At 8.4 W, the bonded region exposed on the fracture surface was relatively limited, consistent with the smaller effective bonded-track width measured in Figure 10. Cleavage steps were observed at different locations on the fracture surface. Points 1 and 3 mainly contained Al and O, with Al:O atomic ratios of approximately 1:1.30 and 1:1.87, respectively. In view of the uncertainty in EDS oxygen quantification noted above, these ratios are not interpreted as exact stoichiometric agreement with α-Al_2_O_3_. Instead, the predominance of Al and O, together with the negligible Ti contents and the corresponding fracture-surface characteristics, supports the assignment of these locations as sapphire-rich fracture regions. In contrast, Points 2 and 4 contained appreciable Ti together with Al and O, indicating that the fracture locally passed through the interfacial compositional transition region. This indicates that the joint exhibited a mixed fracture mode involving both the joint interface and the sapphire substrate. As observed in Figure 12c,f, regions enriched in Al and O occupied only a small area of the fracture surface, indicating that fracture mainly propagated along the joint interface, which agrees well with the fracture-path analysis in Figure 11a.

Figure 12g–l display the fracture microstructure and EDS analysis results of joints welded at a laser power of 12.4 W. Figure 12h,k is magnified views of local regions. The fracture surface was characterized by cleavage fracture, with a marked increase in cleavage steps. As shown in Table 4, Point 7 contained 51.07 at% Al, 33.53 at% Ti, and 14.72 at% O, while Point 10 contained 46.19 at% Al, 33.91 at% Ti, and 18.88 at% O, corresponding to Al:Ti atomic ratios of approximately 1.52:1 and 1.36:1, respectively. The substantial coexistence of Al and Ti at these locations indicates pronounced Al–Ti intermixing and shows that the fracture passed through an Al–Ti-containing interfacial region. These measured Al:Ti ratios, however, do not correspond to the ideal 3:1 stoichiometry of Al_3_Ti and therefore are not used to independently identify this specific phase. The EDS point analyses are used here only to characterize the local elemental composition and elemental intermixing within the fracture region, whereas the specific identification of Al_3_Ti is based on the XRD results shown in Figure 5. The crack propagation direction was repeatedly deflected owing to the wavy interfacial morphology under shear loading, which agrees well with the fracture-path analysis in Figure 11f. Such crack deflection increases the fracture path and promotes energy dissipation, thereby contributing to the higher apparent shear strength at 12.4 W. In addition, numerous fine cleavage steps were observed near Points 6 and 11 in Figure 12h,k, further indicating local deflection of the crack path during shear fracture. These fracture characteristics are consistent with the enhanced apparent shear strength observed at 12.4 W.

Figure 12m–r show the fracture microstructure and EDS analysis results of joints fabricated at a laser power of 16.6 W. Figure 12o,r is magnified views of local regions. The fracture mode remained predominantly cleavage fracture. According to Figure 12o,r and Table 4, Points 12, 14, 15, and 16 exhibited Al:O atomic ratios ranging from approximately 1:1.41 to 1:1.93. Consistent with the EDS oxygen-quantification uncertainty noted above, these ratios are not used as exact stoichiometric criteria for phase identification. Instead, the predominance of Al and O, the negligible Ti contents, and the corresponding fracture morphologies together support the assignment of these locations as sapphire-rich fracture regions, indicating that fracture occurred predominantly on the sapphire side. In contrast, Point 13 contained appreciable Ti together with Al and O, indicating that the fracture locally passed through the interfacial compositional transition region. Large herringbone-shaped cleavage steps were observed on the sapphire substrate near Point 14, which matches the morphology of the fracture path in Figure 11k. Pronounced brittle fracture features were observed in the sapphire substrate near Point 15, corresponding to the missing sapphire region after welding. The high-heat-input condition was accompanied by continuous cracking on the sapphire side, consistent with the enhanced thermal-mismatch stresses discussed above. Such cracks facilitated crack initiation and propagation, thereby contributing to the reduced apparent shear strength.

## 4. Conclusions

In this study, direct joining of sapphire and TC4 alloy was achieved using femtosecond laser welding at a high scanning speed of 200 mm/s. The effects of laser power on joint formation, interfacial structure, metallurgical reactions, and mechanical properties were systematically investigated. The main conclusions are as follows:

(1) Within the laser power range of 8.4–16.6 W, continuous welds were achieved for all sapphire/TC4 alloy joints, and macroscopic cracks were absent in the sapphire substrate. Post-weld sapphire preserved favorable optical transparency, showing no obvious thermal discoloration, haze or through-thickness cracking.
(2) The femtosecond laser induced localized interfacial melting, elemental redistribution, and metallurgical reactions at the sapphire/TC4 alloy interface. The interfacial compositional transition zone reached a maximum width of 5.72 μm. Reaction products including TiO, TiO_2_, and Al_3_Ti were identified by XRD, while EDS analysis revealed interfacial redistribution and intermixing of Ti, Al, and O. Overall, the interfacial structure developed through the coupled effects of localized melting and liquid spreading, elemental transport, interfacial reactions, and rapid solidification.
(3) Thermodynamic analysis showed strong temperature dependence of the candidate interfacial reactions. The high-temperature stage, which may extend above 1400 K, primarily promoted interfacial melting and elemental redistribution. For direct comparison, Reactions (1)–(3) were evaluated on a common Ti-normalized basis over their common applicable range of 500–1400 K. At 1400 K, the Al_3_Ti-forming reaction exhibited the most negative Ti-normalized *ΔG* among Reactions (1)–(3), and its thermodynamic driving force increased further with decreasing temperature within this range. These results, together with the XRD identification of Al_3_Ti, support the formation and retention of Al_3_Ti during rapid cooling.
(4) At 8.4 W, the joint exhibited a relatively flat interface and limited crack deflection, which were associated with a lower apparent shear strength than that at 12.4 W. The highest mean apparent shear strength of 51.55 MPa was obtained at 12.4 W; the characteristic wavy interfacial morphology promoted crack deflection, increased the fracture path, and enhanced energy dissipation, thereby contributing to the higher apparent shear strength. At 16.6 W, excessive heat input was accompanied by continuous cracking within the sapphire and near the interface, which impaired the load-bearing capability of the joint. No significant difference was observed between the apparent shear strengths at 8.4 and 16.6 W (*p* = 0.927), indicating that they were comparable within the experimental scatter, although the underlying limiting mechanisms were different.

## Figures and Tables

**Table 1:** Certified chemical composition (wt%) of the TC4 alloy according to quality certificate JS2023-311.

Materials	Ti	Al	V	Fe	C	O	N	H	Others
TC4 alloy	Bal.	5.88	4.02	0.13	0.05	0.16	0.007	0.015	≤0.10 each ≤0.40 total

**Table 2:** Laser energy characteristics used in the experiments.

Average Power (W)	Single Pulse Energy (μJ)	Single-Pulse Fluence (J/cm^2^)	Linear Energy Density (J/mm)
8.4	10.50	5.942	0.042
12.4	15.50	8.771	0.062
16.6	20.75	11.742	0.083

**Table 3:** Gibbs free-energy changes of the candidate interfacial reactions at 1400 K and the corresponding Ti-normalized values.

Reaction at 1400 K	*ΔG* (kJ per mol of Reaction)	Ti-Normalized *ΔG* (kJ·mol^−1^ Ti)
Al_2_O_3_ (s) + 3Ti (s) → 2Al (l) + 3TiO (s)	−0.086	−0.029
3Al (l) + Ti (s) → Al_3_Ti (s)	−95.326	−95.326
Al (l) + 3Ti (s) → Ti_3_Al (s)	−75.165	−25.055
2TiO (s) + O_2_ (g) → 2TiO_2_ (s)	−563.621	—

*ΔG* is expressed per mole of reaction as written. Reaction (4) was not Ti-normalized because it does not directly consume metallic Ti.

**Table 4:** EDS point-analysis results for the chemical compositions of the fracture regions of sapphire/TC4 alloy joints.

Position	Element (At%)			
	Al	O	Ti	V
1	43.35	56.43	0.23	0.00
2	21.76	68.20	9.74	0.31
3	34.78	65.11	0.07	0.04
4	28.32	60.21	11.12	0.35
5	38.18	61.23	0.38	0.21
6	15.47	76.16	8.15	0.23
7	51.07	14.72	33.53	0.68
8	25.34	63.77	10.53	0.36
9	32.83	54.71	11.97	0.49
10	46.19	18.88	33.91	1.02
11	20.53	68.28	10.94	0.25
12	40.56	59.34	0.11	0.00
13	33.08	55.21	11.69	0.03
14	33.98	65.71	0.29	0.02
15	36.86	62.69	0.38	0.06
16	41.47	58.48	0.04	0.01

## Data Availability

The original contributions presented in this study are included in the article/Supplementary Material. Further inquiries can be directed to the corresponding authors.

## Supplementary Materials

The following supporting information can be downloaded at: https://www.mdpi.com/article/10.3390/ma19204286/s1, Table S1: Individual apparent shear-strength values of the seven valid sapphire/TC4 alloy joints under different laser-power conditions.
