# Supplementary material for: Interfacial Characteristics and Metallurgical Mechanism of Sapphire/TC4 Alloy Joints Produced by Femtosecond Laser Welding
Source: Materials (Basel). 2026 Oct 11;19(20):4286. doi: 10.3390/ma19204286 (PMC13707257; doi:10.3390/ma19204286)
Supplement: Supplementary file 1 [file materials-19-04286-s001.zip › materials-4527912-supplementary.pdf]

Table S1. Individual apparent shear-strength values of the seven valid sapphire/TC4 alloy joints under different laser-power conditions.

Specimen No.	8.4 W (MPa)	12.4 W (MPa)	16.6 W (MPa)
1	30.75096	58.43622	43.85356
2	43.91592	48.90710	34.22022
3	37.42570	50.16435	36.04511
4	31.63210	49.63820	39.57640
5	40.36800	54.12500	32.69240
6	33.32520	47.69310	34.52770
7	35.92460	51.85270	38.17820
**Mean ± SD**	**36.19 ± 4.78**	**51.55 ± 3.69**	**37.01 ± 3.84**

Note: The values correspond to the seven valid shear tests included in the statistical analysis for each laser-power condition. Apparent shear strength was calculated using the estimated effective bonded area for the corresponding laser-power condition. The effective bonded areas were 13.68, 15.84, and 19.44 mm^2 for 8.4, 12.4, and 16.6 W, respectively. The data are reported as mean ± standard deviation (SD), with $n = 7$ for each condition.
